# O Efeito Direto do Índice de Massa Corporal nos Resultados Cardiovasculares entre Participantes sem Obesidade Central pela Estimativa por Máxima Verossimilhança Direcionada

**DOI:** 10.36660/abc.20200231

**Published:** 2021-05-06

**Authors:** Hossein Mozafar Saadati, Siamak Sabour, Mohammad Ali Mansournia, Yadollah Mehrabi, Seyed Saeed Hashemi Nazari

**Affiliations:** 1 Shahid Beheshti University of Medical Sciences Faculdade de Saúde Pública e Segurança Departamento de Epidemiologia Tehran Irã Departamento de Epidemiologia, Faculdade de Saúde Pública e Segurança, Shahid Beheshti University of Medical Sciences, Tehran - Irã.; 2 Tehran University of Medical Sciences Faculdade de Saúde Pública Departamento de Epidemiologia e Bioestatística Tehran Irã Departamento de Epidemiologia e Bioestatística, Faculdade de Saúde Pública, Tehran University of Medical Sciences, Tehran - Irã.; 3 Shahid Beheshti University of Medical Sciences Faculdade de Saúde Pública e Segurança Centro de Pesquisa de Prevenção de Doenças Cardiovasculares Tehran Irã Centro de Pesquisa de Prevenção de Doenças Cardiovasculares, Faculdade de Saúde Pública e Segurança, Shahid Beheshti University of Medical Sciences, Tehran - Irã.

**Keywords:** Obesidade, Doenças Cardiovasculares, Índice de Massa Corporal, Circunferência Abdominal, Circunferência da Cintura, Aterosclerose; Fatores de Risco

## Abstract

**Fundamento:**

O índice de massa corporal (IMC) é o índice mais usado para categorizar uma pessoa como obesa ou não-obesa, e está sujeito a limitações importantes.

**Objetivo:**

Avaliar o efeito direto do IMC nos desfechos cardiovasculares em participantes sem obesidade central.

**Métodos:**

Esta análise incluiu 14.983 homens e mulheres com idades entre 45-75 anos do Estudo de Risco de Aterosclerose em Comunidades (ARIC). O IMC foi medido como obesidade geral e a circunferência da cintura (CC), a relação cintura-quadril (RCQ) e circunferência do quadril como obesidade central. A estimativa de máxima verossimilhança direcionada (TMLE, no acrônimo em inglês) foi usada para estimar os efeitos totais (TEs) e os efeitos diretos controlados (CDEs). A proporção de ET que seria eliminada se todos os participantes fossem não obesos em relação à obesidade central foi calculada usando o índice de proporção eliminada (PE). P<0,05 foi considerado estatisticamente significativo. As análises foram realizadas no pacote TMLE R.

**Resultados:**

O risco de desfechos cardiovasculares atribuídos ao IMC foi significativamente revertido com a eliminação da obesidade na RCQ (p <0,001). A proporção eliminada dos efeitos do IMC foi mais tangível para participantes não obesos em relação à CC (PE = 127%; IC95% (126,128)) e RCQ (PE = 97%; IC95% (96,98)) para doença arterial coronariana (DAC), e RCQ (PE = 92%; IC95% (91,94)) para acidente vascular cerebral, respectivamente. Com relação ao sexo, a proporção eliminada dos efeitos do IMC foi mais tangível para participantes não obesos em relação a RCQ (PE = 428%; IC95% (408.439)) para DAC em homens e CC (PE = 99%; IC95% (89,111)) para acidente vascular cerebral em mulheres, respectivamente.

**Conclusão:**

Esses resultados indicam diferentes efeitos potenciais da eliminação da obesidade central na associação entre IMC e desfechos cardiovasculares em homens e mulheres. (Arq Bras Cardiol. 2021; 116(5):879-886)

## Introdução

A obesidade, como fator preditor de doença cardiovascular, possui várias definições e critérios. O índice de massa corporal (IMC) é o mais usado para classificar uma pessoa como obesa ou não obesa.^1^ No entanto, esse índice está sujeito a limitações importantes,^1^^,^^2^ pois não fornece informações sobre distribuição de gordura, e também não pode discriminar entre as diferentes massas corporais (músculos, ossos e gordura). Essas limitações podem levar a uma classificação incorreta dos níveis de obesidade.^3^^,^^4^ Por outro lado, os índices de obesidade - central, como circunferência da cintura (CC) e relação cintura-quadril (RCQ), como medidas simples e alternativas de obesidade, medem diretamente a massa de gordura central, o que fornece informações importantes sobre os desfechos de saúde.^5^ Em um estudo de coorte, verificou-se que a CC pode nem sempre estar alinhada com o IMC, e foi proposto que uma combinação de IMC e CC poderia estimar melhor as doenças relacionadas à obesidade.^6^ Além disso, o IMC é um índice geral de obesidade e fornece evidências contraditórias entre adultos e pessoas com 65 anos ou mais. Esse fenômeno é conhecido como “paradoxo da obesidade”.^7^^,^^8^

Para revelar uma relação causal, precisamos controlar ao máximo os potenciais confundidores e as suposições causais. A esse respeito, dois métodos causais — Ponderação pela Probabilidade Inversa (IPW – do inglês, Inverse Probability Weighting) e fórmula G — foram introduzidos. Eles são baseados em modelos de exposição e resultados, respectivamente. Em relação ao nosso tópico, se o modelo ajustado for especificado incorretamente, os resultados serão enviesados. Os métodos duplamente robustos têm a vantagem de usar simultaneamente modelos de exposição e de resultado e, se apenas um deles for mal especificado, o resultado ainda é válido.^9^^,^^10^ Considerando as limitações do IMC e as restrições dos estudos observacionais, usamos a estimativa de máxima verossimilhança direcionada (TMLE, Targeted Maximum Likelihood Estimation) como um estimador duplamente robusto para reduzir o viés dos parâmetros-alvo se a exposição ou os mecanismos de resultado fossem estimados de forma consistente,^10^ para estimar os efeitos totais (TEs) e os efeitos diretos controlados (CDEs) do IMC. Portanto, este estudo teve como objetivo determinar os TEs e CDEs do IMC nos desfechos cardiovasculares para demonstrar a importância do efeito total do IMC nos desfechos cardiovasculares e quanto desse efeito seria eliminado se todos os participantes fossem não obesos em relação à obesidade central (CDE).

## Método

### Participantes

O estudo Risco de Aterosclerose em Comunidades (ARIC) foi um estudo de coorte prospectivo que começou em 1987 em quatro condados nos EUA (Washington County, Maryland; Jackson, Mississippi; Forsyth County, Carolina do Norte; e os subúrbios de Minneapolis, Minnesota). Os investigadores recrutaram 15.792 participantes com idades entre 45-64 anos. Mais detalhes são descritos em outros estudos.^11^ Analisamos todos os dados desde a visita um (1987-1989) e a ocorrência do desfecho até 2014. Para o presente estudo, os participantes com informações ausentes ou histórico de qualquer doença cardiovascular foram excluídos. Os conselhos de revisão institucional de cada centro aprovaram o protocolo do estudo ARIC e um termo de consentimento livre e esclarecido foi obtido de todos os participantes em cada visita do estudo.

### Medidas

#### Exposição: obesidade por definição de índice de massa corporal

Neste estudo, a principal exposição de interesse é a obesidade com definição de IMC. O IMC foi calculado como peso em quilogramas dividido a altura em metros ao quadrado. Obesidade geral foi definida como IMC ≥30 kg/m^2^.

#### Mediadores: índices de obesidade central definidos por circunferência da cintura, relação cintura-quadril e circunferência do quadril

Para avaliar o efeito direto controlado do IMC mediado pela obesidade central (massa gorda central), consideramos três definições para obesidade central, incluindo CC, RCQ e circunferência do quadril. A CC foi categorizada no ponto de corte ≥102 cm nos homens e no ponto de corte ≥88 cm nas mulheres. O valor de corte da RCQ foi estabelecido em ≥0,9 para homens e ≥0,85 para mulheres, de acordo com a Organização Mundial da Saúde (OMS).^12^ Uma vez que não existe um acordo universal quanto ao valor de corte da circunferência do quadril, ele foi avaliado com base no melhor valor de limiar em uma curva ROC (Receiver Operating Character).

### Doença coronariana, insuficiência cardíaca, acidente vascular cerebral e mortalidade por todas as causas como resultados

Os resultados deste estudo incluíram eventos de doença arterial coronária (DAC) e insuficiência cardíaca (IC) registrados até 31 de dezembro de 2014. De acordo com os critérios do estudo ARIC, os resultados de DAC são definidos como infarto do miocárdio definitivo ou provável ou DAC fatal. Os resultados da IC são definidos com base nos critérios CID-9 e CID-10. Incidente de IC foi definido como hospitalização que incluiu o código para IC começando com “428” (ou seja, 428,0 a 428,9) em qualquer posição, ou uma certidão de óbito com código CID-9 começando com “428” ou código CID-10 “I50” (IC ou I50.0 a I50.9) em qualquer posição. Os eventos de acidente vascular cerebral (AVC) foram identificados por acompanhamento anual, códigos hospitalares CID-9 430 a 436 (listados como um código de diagnóstico de alta em qualquer posição) ou em certidões de óbito. A mortalidade por causa específica foi classificada com base nos atestados de óbito: mortalidade cardiovascular (códigos CID-9 390-459, códigos CID-10 I00-I99), mortalidade por câncer (códigos CID-9 140-239, códigos CID-10 C00-D49), e todas as outras causas de morte.

### Covariáveis e fatores confundidores

Os dados covariáveis foram considerados fatores de confusão na associação de desfecho de exposição, mediador de exposição e associação mediador-desfecho. Idade, sexo (masculino e feminino), raça (preto e branco), nível de educação (básico, intermediário, avançado), local (condado de Washington, condado de Forsyth, cidade de Jackson, subúrbios do noroeste de Minneapolis selecionados), tabagismo (definido como atual, ex-fumante e nunca fumante), consume de bebidas alcoólicas (definido como consumo atual, ex-alcoólatra e nunca consumiu bebidas alcoólicas) e pontuação total de atividade física (em três dimensões: no trabalho, no lazer e nos esportes) foram baseados em questionários de autorrelato. Outras covariáveis incluíram ingestão total de calorias (kcal), hipertensão (pressão arterial sistólica ≥140 ou pressão arterial diastólica ≥90 mmHg, ou uso de qualquer medicamento para pressão alta), diabetes mellitus (glicemia ≥200 e glicemia em jejum ≥126 mg/dl, ou uso de qualquer medicamento para diabetes), lipídios plasmáticos (mg/dl) e histórico de AVC no início do estudo. Os lipídios plasmáticos incluíam colesterol, colesterol de lipoproteína de alta densidade e triglicerídeos. As covariáveis biológicas foram excluídas das análises de TE e incluídas como potenciais confundidores da associação mediador-desfecho nas análises de CDE.

### Análise estatística

Estatísticas descritivas foram utilizadas para descrever os participantes (média ± desvio-padrão (DP) para variáveis contínuas e número e porcentagem para variáveis categóricas). Uma análise de teste t independente foi usada para examinar as diferenças estatísticas em covariáveis contínuas entre dois níveis de exposição de interesse (IMC). Além disso, o teste *χ*2 foi usado para examinar as associações de variáveis categóricas com a exposição. A normalidade dos dados foi avaliada pela curva normal (assimetria e desvio-padrão da assimetria) e pelo teste de Kolmogorov-Smirnov. Para calcular o efeito direto controlado do IMC sobre os desfechos (CC, IC, AVC e mortalidade por todas as causas) mediados pela obesidade central, foi utilizado o modelo TMLE. O TMLE, como estimador duplamente robusto, usa modelos de resultado e exposição. A implementação do TMLE segue as seguintes etapas: na primeira, geramos estimadores para o modelo de desfecho da exposição e todos os confundidores listados. Em seguida, geramos estimadores para o modelo de tratamento (exposição e desfecho ausente) em todos os fatores de confusão listados. Na terceira etapa, calculamos a covariável inteligente, H, com base no modelo de tratamento (exposição e desfecho ausente) para ambos expostos “H = 1/PS” (PS como escore de propensão, a probabilidade de exposição) e grupos não expostos “H = -1 / (1-PS)”.^10^^,^^13^

O mecanismo ausente é definido como a ocorrência de um evento competitivo (mortalidade total por todas as outras causas, AVC, DAC e IC, para cada desfecho interessado) ou perda de acompanhamento antes da ocorrência do desfecho de interesse, onde “ausente = 1” Indica que o resultado é observado e “ausente = 0” indica que o resultado está ausente. Usamos uma definição dicotômica de exposição (IMC); os valores acima do ponto de corte definido foram classificados como “obesos” e os abaixo do ponto de corte como “não obesos”. Para a variável mediadora, foram utilizados os três índices de obesidade central. Dessa forma, fixamos os valores do mediador em zero (não obesos de acordo com a obesidade central), de acordo com o modelo causal contrafactual, e avaliamos o efeito direto controlado. TE, na abordagem de inferência causal, é normalmente definido como a diferença entre o resultado de interesse de um indivíduo ou grupo, se exposto a uma exposição específica, e o resultado do mesmo indivíduo ou grupo, se não exposto. O CDE é geralmente definido como a diferença entre o resultado de interesse de um indivíduo ou grupo, se exposto a uma exposição específica, e o resultado do mesmo indivíduo ou grupo, se não exposto, ao fixar o valor dos mediadores. Em nosso estudo, o CDE do IMC foi definido como o efeito do IMC após o controle dos índices de CC, RCQ e circunferência do quadril.^14^^,^^15^

Para controlar os fatores confundidores e as possíveis interações, usamos um algoritmo de aprendizagem de máquina de superaprendizado, que modela diferentes combinações de fatores de confusão e que interagem em diferentes modelos, sendo que as estimativas finais são a média ponderada de diferentes estimativas de modelos.

Ajustamos os algoritmos (modelo linear generalizado, MLG stepwise e MLG de interação) para cada um dos modelos de exposição e resultado, inserindo todas as covariáveis listadas como preditoras e o IMC como exposição binária.

Em seguida, calculamos o efeito de tratamento aditivo (ETA) como diferença de risco para TEs e CDEs, e os intervalos de confiança correspondentes. A estimativa de variância baseada na curva de influência foi usada para estimar os intervalos de confiança. A validação interna foi realizada no modelo superaprendizado como validação cruzada. A proporção eliminada foi calculada de acordo com a seguinte fórmula:^16^

$$PE\left(m\right)=\frac{TE-CDE\left(m\right)}{TE}$$

Onde PE é a proporção eliminada, TE é o efeito total, CDE é o efeito direto controlado e m é a fixação do nível do mediador em zero (não obeso). Intervalos de confiança (95%) para PE foram avaliados usando o método *bootstrap*. O valor de P<0,05 foi considerado estatisticamente significativo. A análise foi realizada no pacote TMLE R versão 3.5.3.

## Resultados

### Características dos participantes

Dos 14.983 participantes na linha de base, incluímos 12.085, 12.085, 12.725 e 12.936 participantes nesta análise depois de excluir todos os indivíduos com histórico de qualquer doença cardiovascular e dados ausentes na linha de base para DAC, IC, AVC e mortalidade por todas as causas, respectivamente. Para mortalidade por todas as causas, incluímos todos os participantes com histórico de qualquer doença cardiovascular. Durante uma mediana de 27 anos de acompanhamento, 1.616 (13,37%), 2.229 (18,44%), 1.078 (8,47%) e 5.364 (41,47%) participantes tiveram DAC, IC, AVC e mortalidade por todas as causas, respectivamente. Nesse período, 3.416 (22,8%) e 1.035 (6,91%) participantes tiveram perda de acompanhamento e risco competitivo, respectivamente. Em relação aos participantes com obesidade com base no IMC, durante uma mediana de 27 anos de acompanhamento, 500 (16,43%), 848 (27,86%), 357 (10,67%) e 1.676 (49,08%) participantes experimentaram DAC, IC, AVC e mortalidade por todas as causas, respectivamente. As características da linha de base (média e desvio-padrão para variáveis contínuas e número e porcentagem para variáveis categóricas para participantes com e sem obesidade, com base no IMC) são fornecidas na Tabela 1 e nas Tabelas complementares 1-4. Os participantes obesos, por definição de IMC, eram mais propensos a serem mulheres, de cor parda, ter nível educacional mais baixo e renda familiar anual mais baixa, e menos propensos a terem plano de saúde em comparação com indivíduos não obesos. Em relação às variáveis mediadoras, participantes obesos tinham maior probabilidade de ser obesos com base nos índices de CC, circunferência do quadril e RCQ, respectivamente.

**Tabela 1:** CaracterÍsticas de base dos participantes do Estudo ARIC por IMC, 1987-2014

Características		Índice de massa corporal		Valor de p*
		Obeso	Não obeso	
Confundidores categóricos		No. %		
Sexo	feminino	2.484 (60,85)	5.686 (52,16)	<0,001
	masculino	1.598 (39,15)	5.215 (47,84)	
Raça	branca	2.514 (61,59)	8.613 (79,01)	<0,001
	negra	1.568 (38,41)	2.288 (20,99)	
Educação	Básica	1.237 (30,39)	2.300 (21,12)	<0,001
	Intermediária	1.633 (40,11)	4.494 (41,27)	
	Avançada	1.201 (29,50)	4.094 (37,60)	
Family Income (per year) Renda familiar (por ano)	menos de $16,000	1.208 (31,71)	2.011 (19,48)	<0,001
	$16,000 –$50,000	1.948 (51,13)	5.437 (52,66)	
	mais de $50,000	654 (17,17)	2.876 (27,86)	
Ingestão de bebida alcoólica	Bebe atualmente	1.805 (44,61)	6.591 (60,64)	<0,001
	Bebia no passado	912 (22,54)	1.931 (17,77)	
	Nunca bebeu	1.329 (32,85)	2.347 (21,59)	
Tabagismo	Fumante	793 (19,44)	3.158 (28,99)	<0,001
	Ex-fumante	1.369 (33,56)	3.487 (32,01)	
	Nunca fumou	1.917 (47,00)	4.248 (39,00)	
Plano de saúde	Não	562 (13,82)	901 (8,27)	<0,001
	Sim	3,506 (86,18)	9,991(91,73)	
Histórico familiar de CAD	Não	1,719 (42,70)	4.556 (42,42)	0,76
	Sim	2.307 (57,30)	6.183 (57,58)	
Hipertensão	Não	2.229 (54,97)	8.258 (76,16)	<0,001
	Sim	1.826 (45,03)	2.585 (23,84)	
Remédios para hipertensão	Não	2.208 (54,12)	8.156 (74,85)	<0,001
	Sim	1.872 (45,88)	2.740 (25,15)	
Diabetes mellitus	Não	3.234 (80,31)	10.114 (93,38)	<0,001
	Sim	793 (19,69)	717 (6,62)	
**Confundidores contínuos**		**Média (DP)**		
Idade, anos		54,09 (5,70)	54,30 (5,78)	0,04
Atividade física (trabalho)		2,18 (0,99)	2,17 (0,93)	0,67
Atividade física (esporte)		2,27 (0,72)	2,49 (0,81)	<0,001
Atividade física (lazer)		2,26 (0,57)	2,39 (0,57)	<0,001
Ingestão total de energia (Kcal)		1632,4 (702,3)	1637,2 (703,1)	0,72
Ácidos graxos saturados (% Kcal)		12,23 (2,93)	11,93 (3,02)	<0,001
Colesterol total mg/dl		5,62 (1,12)	5,54 (1,07)	<0,001
Triglicerídeos mg/dl		1,76 (1,28)	1,40 (0,89)	<0,001
Colesterol HDL mg/dl		1,20 (0,36)	1,37 (0,46)	<0,001
**Mediadores**		**No. %**		
Circunferência da cintura	Não obeso	108 (2,65)	6.893 (63,23)	<0,001
	Obeso	3.974 (97,35)	4.008 (36,77)	
Relação cintura-quadril	Não obeso	275 (6,74)	2.927 (26,85)	<0,001
	Obeso	3.807 (93,26)	7.974 (73,15)	
Circunferência do quadril	Não obeso	989 (24,23)	10.246 (93,99)	<0,001
	Obeso	3.093 (75,77)	655 (6,01)	

*Valor de p baseado no teste χ2 e teste t independente para variáveis categóricas e contínuas, respectivamente; ARIC: Estudo de Risco de Aterosclerose em Comunidades; Média e desvio-padrão das variáveis contínuas em cada grupo de índice de massa corporal; Número e porcentagem de variáveis categóricas em cada grupo de índice de massa corporal*.

### Efeitos totais e efeitos diretos controlados

Os TEs e CDEs do IMC, para todos os desfechos de interesse como efeito de tratamento aditivo (diferença de risco) com intervalos de confiança de 95%, estimados por TMLE para todos os participantes e por sexo, estão demonstrados nas Tabelas 2 e 3 e na Figura 1 complementar. Sobre os TEs, os resultados mostram uma associação forte e significativa entre o IMC e todos os resultados. Os mais fortes são estimados para IC, mortalidade por todas as causas, DAC e AVC, respectivamente. Em relação a CDEs, grandes CDEs para IC e mortalidade por todas as causas, após o controle de todos os três índices de obesidade central separadamente (ETA entre = 4,27 e 7,95), sugerem que, mesmo se a obesidade central fosse eliminada, permaneceria um forte efeito para o IMC. Por outro lado, pequenos efeitos diretos controlados para DAC e AVC, após ajuste para todos os três índices de obesidade central separadamente, especialmente CC em casos de CAD e circunferência do quadril para AVC (ETA entre = -2,81 e 3,06), sugerem que, se a obesidade central fosse eliminada, seria eliminado um forte efeito do IMC.

**Tabela 2:** Estimativa do efeito direto controlado do índice de massa corporal para DAC, IC, AVC e mortalidade por todas as causas, de acordo com Obesidade Central (não obeso), participantes do Estudo ARIC, 1987-2014 (caso completo)

Desfechos	Mediador (Índice de obesidade central, não obeso)	Efeito direto controlado	Proporção eliminada	Efeito total (ETA) (IC95%)
		ETA (IC95%)	PE % (IC95%)	
DAC	CC	-2,81 (-5,01, -0,61)	127 (109, 135)	10,47 (7,76, 13,18)
	RCQ	0,62 (-2,58, 3,82)	94 (79, 99)	
	Quadril	3,06 (-1,20, 7,33)	71 (63, 75)	
IC	CC	6,41 (4,09, 8,72)	60 (55,75)	15,92 (13,45, 18,39)
	RCQ	7,95 (4,54, 11,37)	50 (36,52)	
	Quadril	7,23 (3,33,11,13)	54 (57,64)	
AVC	CC	2,11 (-0,06, 4,29)	76 (74,101)	8,32 (6,01, 10,63)
	RCQ	0,69 (-2,34, 3,73)	92 (81,106)	
	Quadril	0,05 (-3,82,3,92)	99 (93,108)	
Mortalidade (todas as causas)	CC	4,88 (2,56, 7,20)	56 (49,73)	11,05 (9,21, 12,88)
	RCQ	4,27 (0,36, 8,17)	61 (52,69)	
	Quadril	5,19 (2,01,8,36)	53 (50,59)	

*ETA: efeito de tratamento aditivo; PE: proporção eliminada; DAC: doença arterial coronária; IC: insuficiência cardíaca; AVC: acidente vascular cerebral; CC: circunferência da cintura; RCQ: relação cintura-quadril; Quadril: circunferência do quadril*.

**Tabela 3:** Estimativa do efeito direto controlado do IMC na DAC, IC, AVC e mortalidade por todas as causas, de acordo com Obesidade Central (não obeso), em homens e mulheres no Estudo ARIC, 1987-2014 (caso completo)

Sexo	Desfechos	Mediador (Índice de obesidade central)	Efeito direto controlado	Proporção eliminada	Efeito total (ETA) (IC95%)
			ETA (IC95%)	PE % (IC95%)	
Masculino	DAC	CC	-4,32 (-7,69, -0,96)	144 (124,161)	9,83 (5,74, 13,92)
		RCQ	-32,28 (-36,07, -28,48)	428 (408,439)	
		Quadril	2,86 (-2,54, 8,26)	71 (62,81)	
	IC	CC	4,94 (1,31, 8,56)	69 (57,79)	15,76 (12,04, 19,49)
		RCQ	-11,20 (-14,98, -7,42)	171 (158,186)	
		Quadril	15,06 (10,32, 19,79)	4 (0,07,11)	
	AVC	CC	1,14 (-1,63, 3,91)	86 (75,107)	8,10 (3,84, 12,37)
		RCQ	-6,57 (-10,63, -2,50)	181 (174,201)	
		Quadril	1,13 (-4,42, 6,69)	86 (76,95)	
	Mortalidade (todas as causas)	CC	5,57 (1,38, 9,77)	49 (37,68)	10,89 (8,12, 13,65)
		RCQ	-23,53 (-27,02, -20,04)	316 (301,329)	
		Quadril	6,00 (1,54, 10,46)	45 (36,57)	
Feminino	DAC	CC	5,02 (3,11, 6,93)	57 (43,69)	11,78 (8,70, 14,86)
		RCQ	1,13 (-2,67, 4,92)	90 (79,103)	
		Quadril	4,68 (-0,25, 9,62)	60 (52,67)	
	IC	CC	11,57 (9,18, 13,96)	30 (15,39)	16,66 (13,79,19,53)
		RCQ	9,36 (4,01, 14,70)	44 (31,52)	
		Quadril	8,28 (3,66, 12,89)	50 (37,61)	
	AVC	CC	0,06 (-1,92, 2,05)	99 (89,111)	7,70 (4,73, 10,68)
		RCQ	0,23 (-4,09, 4,55)	97 (87,113)	
		Quadril	4,04 (0,90, 7,19)	47 (34,63)	
	Mortalidade (todas as causas)	CC	5,24 (2,91, 7,57)	53 (41,66)	11,24 (8,76, 13,73)
		RCQ	5,22 (-0,72, 11,16)	53 (42,64)	
		Quadril	1,67 (-3,43, 6,77)	85 (77,98)	

*ETA: efeito de tratamento aditivo; PE, proporção eliminada; DAC: doença arterial coronariana; IC: insuficiência cardíaca; AVC: acidente vascular cerebral; CC: circunferência da cintura; RCQ: relação cintura-quadril; Quadril: circunferência do quadril*.

Em relação ao sexo, os resultados mostram associação forte e significativa entre o IMC e todos os desfechos, tanto para homens quanto para mulheres. Um forte efeito direto controlado para IC em homens e mulheres, exceto para o RCQ em homens nos índices de obesidade central (ETA entre = 4,94 e 15,06), sugere que, mesmo se a obesidade central fosse eliminada, um grande efeito permaneceria para o IMC. Por outro lado, um leve efeito direto controlado para AVC em homens e mulheres, exceto para a circunferência do quadril em mulheres (ETA entre = -6,27 e 1,14) e para DAC em homens (ETA entre = -32,28 a 2,86) nos índices de obesidade central, sugere que, se a obesidade central fosse eliminada, seria eliminado um forte efeito do IMC e, em alguns casos, o efeito do IMC seria revertido (protetor).

### Proporção Eliminada

O índice PE para TEs do IMC, para todos os resultados de interesse com intervalos de confiança de 95% para todos os participantes e sexos, está listado nas Tabelas 2 e 3. A associação total de IMC com DAC pode ser completamente eliminada pela eliminação do papel da CC, em 127%. Este efeito pode ser reduzido em 94% e 71%, eliminando o papel da RCQ e da circunferência do quadril, respectivamente. Em relação ao AVC, o efeito do IMC poderia ser eliminado ao retirar o papel da circunferência do quadril, em 99%. Com relação à IC e à mortalidade por todas as causas, o papel dos índices de obesidade central na eliminação do efeito do IMC foi um pouco semelhante, entre 50% e 61%. Com relação ao sexo, a associação total do IMC com CC, IC, AVC e mortalidade por todas as causas em homens poderia ser completamente eliminada removendo-se o papel da RCQ, em 428%, 171%, 181% e 316%, respectivamente. Por outro lado, em mulheres, a associação total do IMC com CC, IC, AVC e mortalidade por todas as causas não pôde ser completamente eliminada ao remover o papel de quaisquer índices de obesidade central (entre 30% para índice de CC em IC e 99% para índice de CC em AVC).

## Discussão

Neste grande estudo de coorte baseado em comunidades, os TEs e CDEs de IMC relacionados ao risco de DAC, IC, AVC e mortalidade por todas as causas em participantes sem obesidade central foram avaliados usando o método TMLE. É importante mencionar que consideramos duas limitações comuns do IMC e dos estimadores convencionais, incluindo a capacidade limitada do IMC de distinguir entre massa gorda e massa livre de gordura, o que resulta em classificação incorreta e especificação incorreta do modelo, uma fonte comum de viés em estimativas convencionais.

Em resumo, em comparação com os TEs de IMC, os CDEs de IMC entre os participantes sem obesidade central para todos os desfechos de interesse foram atenuados e próximos a nulo. Esses resultados são mais destacados para DAC e AVC. Esse achado destaca a capacidade dos índices de obesidade central de prever o risco de doenças cardiovasculares e mortalidade por todas as causas. Além disso, em relação aos três índices de obesidade central para todos os participantes, a proporção eliminada dos efeitos do IMC não foi consistente para todos os desfechos. A proporção eliminada dos efeitos do IMC foi mais tangível para o índice RCQ em homens, enquanto os resultados não foram consistentes para mulheres. Em geral, para a maioria dos desfechos, os resultados mostraram que, com a redução ou eliminação da obesidade central com base no índice de RCQ, o efeito do IMC foi completamente ou quase todo removido.

Além disso, esses achados destacam as limitações do IMC em prever o risco cardiovascular como um todo ou com base no sexo. Essa discordância do IMC em relação aos desfechos cardiovasculares foi considerada “o paradoxo da obesidade”. Várias explicações para o paradoxo da obesidade relacionado à associação do IMC com doenças cardiovasculares têm sido relatadas. Uma das mais importantes refere-se ao erro de classificação dos níveis de obesidade com base na definição do IMC^4^. Considerando que o IMC é incapaz de discriminar entre massa gorda, massa muscular e superfície corporal, o efeito do IMC é uma combinação desses três tipos de massa.^17^ Portanto, um IMC mais elevado é um indicador não só de maior quantidade de gordura central e visceral, mas também de maior massa muscular ou periférica (gordura ou osso).

Nas últimas décadas, muitos estudos avaliaram a associação de diferentes distribuições de gordura com doenças cardiovasculares, demonstrando que a gordura corporal, principalmente o excesso de gordura central, independentemente da gordura corporal total, é um importante fator de risco para esses desfechos.^18^^–^^22^ A esse respeito, estudos anteriores mostraram que o excesso de gordura em homens é comumente armazenado nas partes viscerais, enquanto nas mulheres é armazenado em partes subcutâneas periféricas.^23^^,^^24^ Os resultados do presente estudo confirmam os achados anteriores e reforçam a importância da distribuição de gordura para homens e mulheres separadamente.

Em relação ao método estatístico em uso, estudos metodológicos anteriores e originais confirmam a superioridade do método TMLE sobre outras abordagens regularmente utilizadas em estudos observacionais para medir causalidade. Nesse sentido, o método de ponderação pela probabilidade inversa (IPW, Inverse Probability Weighting) resulta em estimativas instáveis na presença de pesos extremos e violações da suposição de positividade. Por outro lado, comparado ao método TMLE, o método G só é realizado com base no modelo de resultado e, se especificado incorretamente, traz estimativas enviesadas. TMLE é um estimador duplamente robusto que permanece consistente se a exposição ou os mecanismos de resultado forem estimados de forma consistente.^9^^,^^10^^,^^25^

Trabalhos anteriores confirmam a utilidade do efeito direto controlado, especialmente na avaliação de políticas.^26^^,^^27^ No entanto, o uso desse conceito necessita de outras premissas que não as TEs.^16^^,^^26^ Na análise direta controlada, deve-se considerar a suposição para a associação entre mediador e desfecho, bem como a associação entre exposição e desfecho.^28^ Além disso, a interação entre a exposição e os mediadores é uma questão importante nesta análise.^16^^,^^28^ Com relação a essa questão, não podemos usar a diferença entre TE e CDE para estimar os efeitos diretos e indiretos.

Em resumo, com base em estudos metodológicos anteriores, no que diz respeito à limitação do efeito direto controlado e à necessidade de premissas mais fortes, isso não pode ser usado como estimativa válida de mediação; mas se tivermos um efeito mediado controlado diferente de zero, pode ser sugestivo da presença de efeito mediador.^28^

Antes de interpretar os resultados e chegar a qualquer conclusão, os pontos fortes e as limitações deste estudo devem ser considerados. Os pontos fortes incluem a aplicação de um método duplamente robusto que estima consistentemente o parâmetro em um modelo semiparamétrico quando um dos dois modelos (exposição e resultado) é especificado corretamente, independentemente de qual. Além disso, consideramos o mecanismo ausente para minimizar o impacto de um risco competitivo e perda de acompanhamento para estimar melhor os efeitos reais. No entanto, devido ao tamanho pequeno da amostra do resultado de interesse e ao viés de dados esparsos, não foi possível avaliar essas estimativas de acordo com faixa etária. Este estudo é limitado, ainda, pelo fato de que não consideramos a variação dos fatores confundidores variáveis no tempo.

## Conclusão

Neste estudo, o efeito direto controlado do IMC caiu para quase nulo em participantes sem obesidade central. Esses resultados destacam a importância de considerar a distribuição das massas de gordura ao estimar a associação entre obesidade e um desfecho de interesse, para homens e mulheres separadamente.

## References

[1] 1. Nuttall FQ. Body mass index: obesity, BMI, and health: a critical review. Nutrition today. 2015;50(3):117. 10.1097/NT.0000000000000092 PMC4890841 27340299

[2] 2. Buss J. Limitations of body mass index to assess body fat. Workplace Health & Safety. 2014;62(6):264 10.1177/216507991406200608 24971823

[3] 3. Frankenfield DC, Rowe WA, Cooney RN, Smith JS, Becker D. Limits of body mass index to detect obesity and predict body composition. Nutrition. 2001;17(1):26-30. 10.1016/s0899-9007(00)00471-8 11165884

[4] 4. Romero-Corral A, Somers VK, Sierra-Johnson J, Thomas RJ, Collazo-Clavell M, Korinek Jec, et al. Accuracy of body mass index in diagnosing obesity in the adult general population. Int J Obes.. 2008;32(6):959-66. 10.1038/ijo.2008.11 PMC2877506 18283284

[5] 5. Lee CMY, Huxley RR, Wildman RP, Woodward M. Indices of abdominal obesity are better discriminators of cardiovascular risk factors than BMI: a meta-analysis.J Clin Epidemiol. 2008;61(7):646-53. 10.1016/j.jclinepi.2007.08.012 18359190

[6] 6. Li C, Ford ES, McGuire LC, Mokdad AH. Increasing trends in waist circumference and abdominal obesity among US adults. Obesity. 2007;15(1):216 10.1038/oby.2007.505 17228050

[7] 7. Chrysant SG, Chrysant GS. The single use of body mass index for the obesity paradox is misleading and should be used in conjunction with other obesity indices. Postgr Med. 2019;131(2):96-102. 10.1080/00325481.2019.1568019 30632444

[8] 8. Iliodromiti S, Celis-Morales CA, Lyall DM, Anderson J, Gray SR, Mackay DF, et al. The impact of confounding on the associations of different adiposity measures with the incidence of cardiovascular disease: a cohort study of 296 535 adults of white European descent. Eur Heart J.2018;39(17):1514-20. 10.1093/eurheartj/ehy057 PMC5930252 29718151

[9] 9. Mansournia MA, Etminan M, Danaei G, Kaufman JS, Collins G. Handling time varying confounding in observational research.BMJ. 2017;359:j4587. 10.1136/bmj.j4587 29038130

[10] 10. Van der Laan MJ, Rose S. Targeted learning: causal inference for observational and experimental data. Philadelphia:Springer Science & Business Media; 2011.

[11] 11. The Atherosclerosis Risk in Communities (ARIC) Study: design and objectives. The ARIC investigators. Am J Epidemiol. 1989;129(4):687-702. 2646917

[12] 12. World Health Organization. Waist circumference and waist-hip ratio Geneva; 2011. (Report of a WHO Expert Consultation Dec.2008)

[13] 13. Almasi-Hashiani A, Nedjat S, Mansournia MA. Causal Methods for Observational Research: A Primer. Arch Iran Med. (AIM). 2018;21(4):164-9. 29693407

[14] 14. Acharya A, Blackwell M, Sen M. Explaining causal findings without bias: Detecting and assessing direct effects. Am Polit Scienc Rev. 2016;110(3):512-29.

[15] 15. Nguyen TQ, Schmid I, Stuart EA. Clarifying causal mediation analysis for the applied researcher: Defining effects based on what we want to learn. arXiv preprint arXiv:190408515. 2019. 10.1037/met0000299 PMC8496983 32673039

[16] 16. VanderWeele T. Explanation in causal inference: methods for mediation and interaction. Oxford: University Press; 2015.

[17] 17. Romero-Corral A, Lopez-Jimenez F, Sierra-Johnson J, Somers VK. Differentiating between body fat and lean mass—how should we measure obesity? Nature Clin & Metab. 2008;4(6):322-3. 10.1038/ncpendmet0809 18382423

[18] 18. Browning LM, Hsieh SD, Ashwell M. A systematic review of waist-to-height ratio as a screening tool for the prediction of cardiovascular disease and diabetes: 0· 5 could be a suitable global boundary value. Nutrit Res Rev. 2010;23(2):247-69. 10.1017/S0954422410000144 20819243

[19] 19. Leitzmann MF, Moore SC, Koster A, Harris TB, Park Y, Hollenbeck A, et al. Waist circumference as compared with body-mass index in predicting mortality from specific causes. PloS one. 2011;6(4):18.582. 10.1371/journal.pone.0018582 PMC3082527 21541313

[20] 20. Shields M, Tremblay MS, Connor Gorber S, Janssen I. Abdominal obesity and cardiovascular disease risk factors within body mass index categories. Health Rep. 2012;23(2):7-15. 22866535

[21] 21. Staiano A, Reeder B, Elliott S, Joffres M, Pahwa P, Kirkland S, et al. Body mass index versus waist circumference as predictors of mortality in Canadian adults. Int J Obes. 2012;36(11):1450-4. 10.1038/ijo.2011.268 PMC4120111 22249224

[22] 22. Van Dijk S, Takken T, Prinsen E, Wittink H. Different anthropometric adiposity measures and their association with cardiovascular disease risk factors: a meta-analysis. Netherl Heart J. 2012;20(5):208-18. 10.1007/s12471-011-0237-7 PMC3346869 22231153

[23] 23. Clark AL, Chyu J, Horwich TB. The obesity paradox in men versus women with systolic heart failure. Am J Cardiol. 2012;110(1):77-82. 10.1016/j.amjcard.2012.02.050 PMC3377856 22497678

[24] 24. Lemieux S, Prud’homme D, Bouchard C, Tremblay A, Després J-P. Sex differences in the relation of visceral adipose tissue accumulation to total body fatness. Am J Clin Nutr. 1993;58(4):463-7. 10.1093/ajcn/58.4.463 8379501

[25] 25. Pang M, Schuster T, Filion KB, Schnitzer ME, Eberg M, Platt RW. Effect Estimation in Point-Exposure Studies with Binary Outcomes and High-Dimensional Covariate Data–A Comparison of Targeted Maximum Likelihood Estimation and Inverse Probability of Treatment Weighting. Int J Biostat. 2016;12(2).Doi:10.1515/ijb-2015-0034 10.1515/ijb-2015-0034 PMC5777857 27889705

[26] 26. Pearl J. Direct and indirect effects. In: Proceedings of the Seventeenth Conference on Uncertainty and Artificial Intelligence; San Francisco: Morgan Kaufmann; 2001;p.411-20.

[27] 27. Robins JM. Semantics of causal DAG models and the identification of direct and indirect effects. Oxford Statistical Science Series. 2003:70-82.

[28] 28. VanderWeele TJ. Controlled direct and mediated effects: definition, identification and bounds. Scand J Stat. 2011;38(3):551-63. 10.1111/j.1467-9469.2010.00722.x PMC4193506 25309023

