# Supplementary material for: O Efeito Direto do Índice de Massa Corporal nos Resultados Cardiovasculares entre Participantes sem Obesidade Central pela Estimativa por Máxima Verossimilhança Direcionada
Source: Arq Bras Cardiol. 2021 May 6;116(5):879–86. [Article in Portuguese] doi: 10.36660/abc.20200231 (PMC8121468; doi:10.36660/abc.20200231)
Supplement: Translation

# Translation: The Direct Effect of Body Mass Index on Cardiovascular Outcomes among Participants Without Central Obesity by Targeted Maximum Likelihood Estimation

**Authors:** Hossein Mozafar Saadati, Siamak Sabour, Mohammad Ali Mansournia, Yadollah Mehrabi, Seyed Saeed Hashemi Nazari

**Affiliations:** 1 Tehran Iran Department of Epidemiology, School of Public Health and safety, Shahid Beheshti University of Medical Sciences, Tehran - Iran.; 2 Tehran Iran Department of Epidemiology and Biostatistics, School of Public Health, Tehran University of Medical Sciences, Tehran - Iran.; 3 Tehran Iran Prevention of Cardiovascular Disease Research Center, School of Public Health and Safety, Shahid Beheshti University of Medical Sciences, Tehran - Iran.

**Keywords:** Body mass index, central obesity, cardiovascular, controlled direct effect, proportion eliminated

## Abstract

**Background:**

Body mass index (BMI) is the most commonly used index to categorize a person as obese or non-obese, which is subject to important limitations.

**Objective:**

To evaluate the direct effect of BMI on cardiovascular outcomes among participants without central obesity.

**Methods:**

This analysis included 14,983 males and females aged 45-75 years from the Atherosclerosis Risk in Communities Study (ARIC). BMI was measured as general obesity, and waist circumference (WC), waist-to-hip ratio (WHR) and hip circumference as central obesity. Targeted maximum likelihood estimation (TMLE) was used to estimate the total effects (TEs) and the controlled direct effects (CDEs). The proportion of TE that would be eliminated if all participants were non-obese regarding central obesity was computed using the proportion eliminated (PE) index. P <0.05 was considered statistically significant. Analyses were performed in the TMLE R package.

**Results:**

The risk of cardiovascular outcomes attributed to BMI was significantly reversed by eliminating WHR obesity (p<0.001). The proportion eliminated of BMI effects was more tangible for non-obese participants regarding WC (PE=127%; 95%CI (126,128)) and WHR (PE=97%; 95%CI (96,98)) for coronary heart disease (CHD), and WHR (PE=92%; 95%CI (91,94)) for stroke, respectively. With respect to sex, the proportion eliminated of BMI effects was more tangible for non-obese participants regarding WHR (PE=428%; 95%CI (408,439)) for CHD in males, and WC (PE=99%; 95%CI (89,111)) for stroke in females, respectively.

**Conclusion:**

These results indicate different potential effects of eliminating central obesity on the association between BMI and cardiovascular outcomes for males and females. (Arq Bras Cardiol. 2021; 116(5):879-886)

## Introduction

Obesity, as a predictor of cardiovascular disease, has several definitions and criteria. Body mass index (BMI) is the most commonly used index to categorize a person as obese or non-obese.^1^ However, this index is subject to important limitations,^1^^,^^2^ as it gives no information regarding fat distribution, and also cannot discriminate between different body masses (muscles, bones and fat). These limitations may lead to a misclassification of obesity levels.^3^^,^^4^ On the other hand, central obesity indices, such as waist circumference (WC) and waist-to-hip ratio (WHR), as simple and alternative measures of obesity, directly measure the central fat mass that gives important information on health outcomes.^5^ In a cohort study, it was found that WC may not always be aligned with BMI and it was proposed that a combination of BMI and WC could provide a better estimate of obesity-related diseases.^6^ In addition, BMI is a general obesity index and provides contradictory evidence among adults and people aged 65 years and older. This phenomenon is known as “the obesity paradox”.^7^^,^^8^

In order to reveal a causal relationship, we need to maximally control the potential confounders and causal assumptions. In this regard, two causal methods—inverse probability weighting (IPW) and G-formula—have been introduced. They are based on exposure and outcome models, respectively. Regarding this issue, if the fitted model is misspecified, the results would then be biased. Double-robust methods have the advantage of simultaneously using both exposure and outcome models, and, if only one of them is misspecified, the result is still valid.^9^^,^^10^ Considering the limitations of BMI and the constraints of observational studies, we used the targeted maximum likelihood estimation (TMLE) as a double-robust estimator to reduce the bias of the target parameters if either exposure or outcome mechanisms are estimated consistently,^10^ aiming to estimate the total effects (TEs) and the controlled direct effects (CDEs) of BMI. Therefore, this study aimed to determine the TEs and CDEs of BMI on cardiovascular outcomes to demonstrate how important the total effect of BMI on cardiovascular outcomes is and how much of this effect would be eliminated if all participants were non-obese with regard to central obesity (CDE).

## Method

### Participants

The Atherosclerosis Risk in Communities (ARIC) study is a prospective cohort study which began in 1987 in four counties in the USA (Washington County, Maryland; Jackson, Mississippi; Forsyth County, North Carolina; and the suburbs of Minneapolis, Minnesota). Investigators recruited 15,792 participants aged 45-64 years. More details are described elsewhere.^11^ We analyzed all data of visit one (1987-1989) and outcome occurrence until 2014. For the present study, participants with missing information or history of any previous cardiovascular disease were excluded. The institutional review boards in each site approved the ARIC study protocol and an informed consent form was obtained from all participants in each study visit.

### Measurements

#### Exposure: Obesity with Body Mass Index Definition

In this study, the main exposure of interest is obesity with BMI definition. BMI was calculated as weight in kilograms divided by the square of height in meters. General obesity was defined as BMI ≥30 kg/m^2^.

#### Mediators: Central Obesity Indices Defined by Waist Circumference, Waist-To-Hip Ratio and Hip Circumference

For evaluating the controlled direct effect of BMI mediated by central obesity (central fat mass), we considered three definitions for central obesity, including WC, WHR and hip circumference. WC was categorized at the cut-off point of ≥102 cm in men and at the cut-off point of ≥88 cm in women. The WHR cut-off value was set at ≥0.9 in men and ≥0.85 in women, according to the World Health Organization (WHO).^12^ Since there is no universal agreement regarding the hip circumference cut-off value, it was evaluated based on the best threshold value in a receiver operating characteristic (ROC) curve.

### Coronary Heart Disease, Heart Failure, Stroke and All-Cause Mortality as Outcomes

The outcomes of this study included coronary heart disease (CHD) and heart failure (HF) events registered by 31 December 2014. According to the ARIC study criteria, CHD outcomes are defined as definite or probable myocardial infarction or fatal CHD. The HF outcomes are defined based on ICD-9 and ICD-10 criteria. Incident HF was defined as hospitalization that included code for HF beginning with “428” (i.e., 428.0 to 428.9) in any position, or a death certificate ICD-9 code beginning with “428” or ICD-10 code “I50” (HF or I50.0 to I50.9) in any position. Stroke events were identified by annual follow-up, hospital ICD-9 codes 430 to 436 (listed as a discharge diagnostic code at any position), or in death certificates. Cause-specific mortality was classified based on death certificates: cardiovascular mortality (ICD-9 codes 390–459, ICD-10 codes I00–I99), cancer mortality (ICD-9 codes 140–239, ICD-10 codes C00–D49), and all other causes of death.

### Covariates and Confounders

Covariate data were derived as confounders in exposure-outcome, exposure-mediator and mediator-outcome association. Age, gender (male and female), race (black and white), education level (basic, intermediate, advanced), center (Washington County, Forsyth County, the city of Jackson, selected northwestern suburbs of Minneapolis), cigarette smoking status (defined as current, former and never smoker), drinking status (defined as current, former and never drinking alcoholic beverages), and total physical activity score (in three dimensions: at work, during leisure time and sports) were based on self-reported questionnaires. Other covariates included total calorie intake (kcal), hypertension (systolic blood pressure ≥140 or diastolic blood pressure ≥90 mmHg, or use of any medication for high blood pressure), diabetes mellitus (blood glucose ≥200 and fasting blood glucose ≥126 mg/dl, or taking any medication for diabetes), plasma lipids (mg/dl) and history of stroke at the baseline. The plasma lipids included cholesterol, high-density lipoprotein cholesterol, and triglycerides. The biologic covariates were excluded from TE analyses and were included as potential confounders of the mediator-outcome association in CDE analyses.

### Statistical Analysis

Descriptive statistics were used to describe the participants (mean ± standard deviation (SD) for continuous variables, and number and percentage for categorical variables). An independent t-test analysis was used to examine the statistical differences in continuous covariates between two levels of exposure of interest (BMI). In addition, the *χ*2 test was used to examine the associations of categorical variables with exposure. The normality of data was evaluated by the normal curve (skewness and standard deviation of skewness) and the Kolmogorov-Smirnov test. To calculate the controlled direct effect of BMI on the outcomes (CHD, HF, stroke and all-cause mortality) mediated through central obesity, the TMLE model was used. TMLE, as a double-robust estimator, uses both outcome and exposure models. The implementation of TMLE has the following steps: in the first step, we generated estimators for outcome model on exposure and all listed confounders. Then, we generated estimators for treatment model (both exposure and missing outcome) on all listed confounders. In the third step, we calculated the clever covariate, H, based on treatment model (both exposure and missing outcome) for both exposed “H = 1/PS” (PS as propensity score, the probability of exposure) and unexposed “H = -1/(1 - PS)” groups.^10^^,^^13^

The missing mechanism is defined as the occurrence of a competing event (total mortality of all other causes, stroke, CHD and HF, for each interested outcome) or loss to follow-up before occurrence of the outcome of interest, where “missing = 1” indicates the outcome is observed, and “missing = 0” indicates the outcome is missing. We used a dichotomous definition of exposure (BMI); values above the defined cut-off point were classified as “obese” and those below the cut-off as “non-obese”. For the mediator variable, the three central obesity indices were used. In this way, we fixed the mediator values to zero (non-obese according to central obesity), according to the counterfactual causal model, and evaluated the controlled direct effect. TE, in the causal inference approach, is often defined as the difference between the outcome of interest of an individual or a group if exposed to a specific exposure, and the outcome of the same individual or group if unexposed. The CDE is often defined as the difference between the outcome of interest of an individual or a group if exposed to a specific exposure, and the outcome of the same individual or group if unexposed while fixing the value of the mediators. In our study, CDE of BMI was defined as the effect of BMI after controlling for WC, WHR and hip circumference indices.^14^^,^^15^

To control the confounders and possible interactions, we used a super learner machine-learning algorithm, which models different combinations of confounders and interactors in different models, and the final estimates are the weighted average of different model estimates.

We fitted the algorithms (generalized linear model, stepwise GLM, and interaction GLM) for each of the exposure and outcome models, inserting all listed covariates as predictors and BMI as binary exposure.

We then calculated the additive treatment effect (ATE) as risk difference for TEs and CDEs, and the corresponding confidence intervals. Influence-curve-based variance estimation was used to estimate the confidence intervals. Internal validation was performed in the super learner model as cross-validation. The proportion eliminated was calculated according to the following formula:^16^

$$PE\left(m\right)=\frac{TE-CDE\left(m\right)}{TE}$$

Where PE is proportion eliminated, TE is total effect, CDE is controlled direct effect and m is fixing the mediator level to zero (non-obese). Confidence intervals (95%) for PE were assessed using the bootstrap method. The value of P < 0.05 was considered statistically significant. The analysis was performed in the TMLE R package version 3.5.3.

## Results

### Participants’ Characteristics

Out of the 14,983 participants at the baseline, we included 12,085, 12,085, 12,725 and 12,936 participants in this analysis after excluding all subjects with a history of any cardiovascular disorder and missing data at the baseline for CHD, HF, stroke and all-cause mortality, respectively. For all-cause mortality, we included all participants with a history of any cardiovascular disorder. During a median 27 years of follow-up, 1,616 (13.37%), 2,229 (18.44%), 1,078 (8.47%) and 5,364 (41.47%) participants experienced CHD, HF, stroke and all-cause mortality, respectively. Within this timeframe, 3,416 (22.8%) and 1,035 (6.91%) participants experienced administrative loss to follow-up and competing risk, respectively. Regarding the participants with obesity based on BMI, during a median 27 years of follow-up, 500 (16.43%), 848 (27.86%), 357 (10.67%) and 1,676 (49.08%) participants experienced CHD, HF, stroke and all-cause mortality, respectively. Baseline characteristics (mean and standard deviation for continuous variables, and number and percentage for categorical variables for participants with and without obesity based on body mass index) are provided in Table 1 and supplementary Tables 1-4. Obese participants by BMI definition were more likely to be females, black-skinned, to have lower educational level, lower annual family income, and were less likely to have health insurance compared to non-obese individuals. Regarding the mediator variables, obese participants were more likely to be obese based on WC, hip circumference and WHR indices, respectively.

**Table 1:** Baseline characteristics of participants in the ARIC Study by BMI, 1987-2014

Characteristics		Body mass index		p-value*
		Obese	Non-obese	
Categorical confounders		No. %		
Sex	female	2,484 (60.85)	5,686 (52.16)	<0.001
	male	1,598 (39.15)	5,215 (47.84)	
Race	white	2,514 (61.59)	8,613 (79.01)	<0.001
	black	1,568 (38.41)	2,288 (20.99)	
Education	Basic	1,237 (30.39)	2,300 (21.12)	<0.001
	Intermediate	1,633 (40.11)	4,494 (41.27)	
	Advanced	1,201 (29.50)	4,094 (37.60)	
Family Income (per year)	Less than $16,000	1,208 (31.71)	2,011 (19.48)	<0.001
	$16,000 –$50,000	1,948 (51.13)	5,437 (52.66)	
	More than $50,000	654 (17.17)	2,876 (27.86)	
Drinking	Current drinker	1,805 (44.61)	6,591 (60.64)	<0.001
	Former drinker	912 (22.54)	1,931 (17.77)	
	Never been a drinker	1,329 (32.85)	2,347 (21.59)	
Smoking	Current smoker	793 (19.44)	3,158 (28.99)	<0.001
	Former smoker	1,369 (33.56)	3,487 (32.01)	
	Never been a smoker	1,917 (47.00)	4,248 (39.00)	
Health insurance	No	562 (13.82)	901 (8.27)	<0.001
	Yes	3,506 (86.18)	9,991(91.73)	
Family history of CVD	No	1,719 (42.70)	4,556 (42.42)	0.76
	Yes	2,307 (57.30)	6,183 (57.58)	
Hypertension	No	2,229 (54.97)	8,258 (76.16)	<0.001
	Yes	1,826 (45.03)	2,585 (23.84)	
Antihypertensive medicine	No	2,208 (54.12)	8,156 (74.85)	<0.001
	Yes	1,872 (45.88)	2,740 (25.15)	
Diabetes mellitus	No	3,234 (80.31)	10,114 (93.38)	<0.001
	Yes	793 (19.69)	717 (6.62)	
**Continual confounders**		**Mean (SD)**		
Age, years		54.09 (5.70)	54.30 (5.78)	0.04
Physical activity (work)		2.18 (0.99)	2.17 (0.93)	0.67
Physical activity (sport)		2.27 (0.72)	2.49 (0.81)	<0.001
Physical activity (leisure time)		2.26 (0.57)	2.39 (0.57)	<0.001
Total energy intake (Kcal)		1632.4 (702.3)	1637.2 (703.1)	0.72
Saturated fatty acid (%Kcal)		12.23 (2.93)	11.93 (3.02)	<0.001
Total cholesterol mg/dl		5.62 (1.12)	5.54 (1.07)	<0.001
Triglycerides mg/dl		1.76 (1.28)	1.40 (0.89)	<0.001
HDL cholesterol mg/dl		1.20 (0.36)	1.37 (0.46)	<0.001
**Mediators**		**No. %**		
Waist circumference	Non-obese	108 (2.65)	6,893 (63.23)	<0.001
	Obese	3,974 (97.35)	4,008 (36.77)	
Waist-to-hip ratio	Non-obese	275 (6.74)	2,927 (26.85)	<0.001
	Obese	3,807 (93.26)	7,974 (73.15)	
Hip circumference	Non-obese	989 (24.23)	10,246 (93.99)	<0.001
	Obese	3,093 (75.77)	655 (6.01)	

*P-value was based on the χ2 test and independent t-test for categorical and continues variables, respectively; ARIC: Atherosclerosis Risk in Communities Study; Mean and standard deviation of continuous variables in each group of body mass index; Number and percentage of categorical variables in each group of body mass index.*.

### Total Effects and Controlled Direct Effects

The TEs and CDEs of BMI, for all outcomes of interest as additive treatment effect (risk difference) with 95% confidence intervals, estimated by TMLE for all participants and sex groups, are demonstrated in Tables 2 and 3 and supplementary Figure 1. Regarding TEs, the results show a strong and significant association between BMI and all outcomes. The stronger results are estimated for HF, all-cause mortality, CHD and stroke, respectively. Regarding CDEs, large CDEs for HF and all-cause mortality, after controlling for all three central obesity indices separately (ATE between = 4.27 and 7.95), suggest that even if central obesities were eliminated, a large effect would remain for BMI. On the other hand, small controlled direct effects for CHD and stroke, after controlling for all three central obesity indices separately, especially for WC in CHD and hip circumference in stroke (ATE between = -2.81 and 3.06), suggest that if central obesities were eliminated, a large effect would be eliminated for BMI.

**Table 2:** Estimated controlled direct effect of body mass index on CHD, HF, Stroke and all-cause mortality, by Central Obesity (non-obese), participants in the ARIC Study, 1987–2014 (complete case)

Outcomes	Mediator (Central Obesity index, non-obese)	Controlled direct effect	Proportion eliminated	Total effect (ATE) (95%CI)
		ATE (95% CI)	PE % (95% CI)	
CHD	WC	-2.81 (-5.01, -0.61)	127 (109, 135)	10.47 (7.76, 13.18)
	WHR	0.62 (-2.58, 3.82)	94 (79, 99)	
	Hip	3.06 (-1.20, 7.33)	71 (63, 75)	
HF	WC	6.41 (4.09, 8.72)	60 (55,75)	15.92 (13.45, 18.39)
	WHR	7.95 (4.54, 11.37)	50 (36,52)	
	Hip	7.23 (3.33,11.13)	54 (57,64)	
Stroke	WC	2.11 (-0.06, 4.29)	76 (74,101)	8.32 (6.01, 10.63)
	WHR	0.69 (-2.34, 3.73)	92 (81,106)	
	Hip	0.05 (-3.82,3.92)	99 (93,108)	
Mortality (all-cause)	WC	4.88 (2.56, 7.20)	56 (49,73)	11.05 (9.21, 12.88)
	WHR	4.27 (0.36, 8.17)	61 (52,69)	
	Hip	5.19 (2.01,8.36)	53 (50,59)	

*ATE: additive treatment effect; PE: proportion eliminated; CHD: coronary heart disease; HF: heart failure; WC: waist circumference; WHR: waist-to-hip ratio; Hip: hip circumference.*.

**Table 3:** Estimated controlled direct effect of BMI on CHD, HF, stroke and all-cause mortality, by Central Obesity (non-obese), in males and females in the ARIC Study, 1987–2014 (complete case)

Sex	Outcomes	Mediator (Central Obesity index)	Controlled direct effect	Proportion eliminated	Total effect (ATE) (95%CI)
			ATE (95% CI)	PE % (95% CI)	
Males	CHD	WC	-4.32 (-7.69, -0.96)	144 (124,161)	9.83 (5.74, 13.92)
		WHR	-32.28 (-36.07, -28.48)	428 (408,439)	
		Hip	2.86 (-2.54, 8.26)	71 (62,81)	
	HF	WC	4.94 (1.31, 8.56)	69 (57,79)	15.76 (12.04,19.49)
		WHR	-11.20 (-14.98, -7.42)	171 (158,186)	
		Hip	15.06 (10.32, 19.79)	4 (0.07,11)	
	Stroke	WC	1.14 (-1.63, 3.91)	86 (75,107)	8.10 (3.84, 12.37)
		WHR	-6.57 (-10.63, -2.50)	181 (174,201)	
		Hip	1.13 (-4.42, 6.69)	86 (76,95)	
	Mortality (all-cause)	WC	5.57 (1.38, 9.77)	49 (37,68)	10.89 (8.12, 13.65)
		WHR	-23.53 (-27.02, -20.04)	316 (301,329)	
		Hip	6.00 (1.54, 10.46)	45 (36,57)	
Females	CHD	WC	5.02 (3.11, 6.93)	57 (43,69)	11.78 (8.70, 14.86)
		WHR	1.13 (-2.67, 4.92)	90 (79,103)	
		Hip	4.68 (-0.25, 9.62)	60 (52,67)	
	HF	WC	11.57 (9.18, 13.96)	30 (15,39)	16.66 (13.79,19.53)
		WHR	9.36 (4.01, 14.70)	44 (31,52)	
		Hip	8.28 (3.66, 12.89)	50 (37,61)	
	Stroke	WC	0.06 (-1.92, 2.05)	99 (89,111)	7.70 (4.73, 10.68)
		WHR	0.23 (-4.09, 4.55)	97 (87,113)	
		Hip	4.04 (0.90, 7.19)	47 (34,63)	
	Mortality (all-cause)	WC	5.24 (2.91, 7.57)	53 (41,66)	11.24 (8.76,13.73)
		WHR	5.22 (-0.72, 11.16)	53 (42,64)	
		Hip	1.67 (-3.43, 6.77)	85 (77,98)	

*ATE: additive treatment effect; PE: proportion eliminated; CHD: coronary heart disease; HF: heart failure; WC: waist circumference; WHR: waist-to-hip ratio; Hip: hip circumference.*.

Regarding sex, the results show strong and significant association between BMI and all outcomes for both males and females. Large controlled direct effect for HF in males and females, except for WHR index in males for central obesity indices (ATE between = 4.94 and 15.06), suggests that even if central obesities were eliminated, a large effect would remain for BMI. On the other hand, small controlled direct effect for stroke in males and females, except for the hip circumference in females (ATE between = -6.27 and 1.14) and for CHD in males (ATE between = -32.28 to 2.86) for central obesity indices, suggests that if central obesities were eliminated, a large effect would be eliminated for BMI, and in some cases the effect of BMI would be reversed (protective).

### Proportion Eliminated

The PE index for TEs of BMI, for all outcomes of interest with 95% confidence intervals for all participants and sex groups, is listed in Tables 2 and 3. The total association of BMI with CHD could be completely eliminated by eliminating the role of WC, in 127%. This effect could be reduced by 94% and 71% by eliminating the role of WHR and hip circumference, respectively. Regarding stroke, the effect of BMI could be eliminated by eliminating the role of hip circumference, in 99%. With respect to HF and all-cause mortality, the role of central obesity indices in eliminating the effect of BMI was somewhat similar and between 50% and 61%. With respect to sex, the total association of BMI with CHD, HF, stroke and all-cause mortality in males could be completely eliminated by eliminating the role of WHR, in 428%, 171%, 181% and 316%, respectively. On the other hand, in females, the total association of BMI with CHD, HF, stroke and all-cause mortality could not be completely eliminated by eliminating the role of any central obesity indices (between 30% for WC index for HF and 99% for WC index for stroke).

## Discussion

In this large, community-based cohort study, the TEs and CDEs of BMI related to the risk of CHD, HF, stroke and all-cause mortality in participants without central obesity were evaluated using the TMLE method. It is worth mentioning that we considered two common limitations of BMI and conventional estimators, including limited capacity of BMI to distinguish between fat mass and fat-free mass, which result in misclassification, and model misspecification, which is a common source of bias in conventional estimators.

In brief, compared to TEs of BMI, the CDEs of BMI among participants without central obesity for all outcomes of interest were attenuated and close to null. Remarkably, these results are more highlighted for CHD and stroke. This finding highlights the capability of central obesity indices to predict the risk of cardiovascular disease and all-cause mortality. In addition, with regard to the three central obesity indices for all participants, the proportion eliminated of BMI effects did not have consistent results for all outcomes. The proportion eliminated of BMI effects was more tangible for WHR index in males, while results were not consistent for females. In general, for most of the outcomes, the results showed that, with reduction or elimination of central obesity based on WHR index, the effect of BMI was completely or mostly removed.

Furthermore, these findings highlighted the limitations of BMI in predicting cardiovascular risk as a whole or based on sex. This disagreement of BMI in relation to cardiovascular outcomes was considered as “the obesity paradox”. Several explanations for the obesity paradox related to the association of BMI with cardiovascular disease have been reported. One of the most important explanations refers to the misclassification of obesity levels based on BMI definition^4^. Considering that BMI is unable to discriminate between fat mass, muscle mass and body surface area, the effect of BMI refers to a combination of these three types of mass.^17^ Therefore, a higher BMI is an indicator not only of greater amount of central and visceral fat, but also of higher muscle or peripheral mass (fat or bone).

In recent decades, many studies have evaluated the association of different fat distributions and cardiovascular disease, demonstrating that the distribution of body fat, especially excess central fat, independent from total body fat, is an important risk factor for these outcomes.^18^^–^^22^ In this regard, previous studies have shown that excessive fat in males is commonly stored in visceral parts, while in females it is stored in peripheral subcutaneous parts.^23^^,^^24^ The results of the present study confirm the previous findings and underline the importance of fat distribution for males and females separately.

Regarding the statistical method in use, previous methodological and original studies confirm the superiority of the TMLE method over other approaches regularly used in observational studies to measure causality. In this regard, the inverse probability weighting (IPW) method result in unstable estimates in the presence of extreme weights and violations of positivity assumption. On the other hand, compared to the TMLE method, the G-formula method is only performed based on the outcome model and, if misspecified, it brings biased estimations. TMLE is a double-robust estimator that remains consistent if either exposure or outcome mechanisms are estimated consistently.^9^^,^^10^^,^^25^

Previous papers confirm the usefulness of the controlled direct effect, especially in policy assessment.^26^^,^^27^ However, the use of this concept needs assumptions other than TEs.^16^^,^^26^ In controlled direct analysis, one must consider the assumption for the association between mediator and outcome as well as the association between exposure and outcome.^28^ In addition, the interaction between exposure and mediators is an important issue in this analysis.^16^^,^^28^ Regarding this issue, we cannot use the difference between TE and CDE to estimate the direct and indirect effects.

In summary, based on previous methodological studies, regarding the limitation of the controlled direct effect and the need for stronger assumptions, this cannot be used as a valid estimation of mediation; but if we have a non-zero controlled mediated effect, it can be suggestive of the presence of mediator effect.^28^

Before interpreting the results and concluding anything, the strengths and limitations of this study should be addressed. The strengths of this study include the application of a double-robust method that consistently estimates the parameter under a semiparametric model when one of two (exposure and outcome) models is correctly specified, regardless of which. In addition, we consider the missing mechanism to minimize the impact of a competing risk and loss to follow-up for better estimation of true effects. However, due to the problem of small sample size of the outcome of interest and sparse-data bias, we could not evaluate these estimations in age groups. In addition, this study is limited by the fact that we did not consider the variation of time-varying confounders.

## Conclusion

In this study, the controlled direct effect of BMI decreased to almost null in participants without central obesity. These results highlight the importance of considering the distribution of fat masses when estimating the association between obesity and an outcome of interest, for males and females separately.
